# A Multi-Channel Urine Sensing Detection System Based on Creatinine, Uric Acid, and pH

**DOI:** 10.3390/bios14100473

**Published:** 2024-10-02

**Authors:** Qiya Gao, Jie Fu, Fangying Xiong, Jiawang Wang, Ziyue Qin, Shuang Li

**Affiliations:** Academy of Medical Engineering and Translational Medicine, Medical College, Tianjin University, Tianjin 300072, China; 3019207445@tju.edu.cn (Q.G.); fujie314159@tju.edu.cn (J.F.); x_0fangying@tju.edu.cn (F.X.); wangjiawang@tju.edu.cn (J.W.); qinziyue@tju.edu.cn (Z.Q.)

**Keywords:** electrochemical sensors, creatinine, uric acid, pH, intelligent medical detection

## Abstract

Urine analysis represents a crucial diagnostic technique employed in clinical laboratories. Creatinine and uric acid in urine are essential biomarkers in the human body and are widely utilized in clinical analysis. Research has demonstrated a correlation between the normal physiological concentrations of creatinine and uric acid in urine and an increased risk of hypertension, cardiovascular diseases, and kidney disease. Furthermore, the pH of urine indicates the body’s metabolic processes and homeostatic balance. In this study, an integrated multi-channel electrochemical sensing system was developed, combining electrochemical analysis techniques, microelectronic design, and nanomaterials. The architecture of an intelligent medical detection system and the production of an interactive interface for smartphones were accomplished. Initially, multi-channel selective electrodes were designed for creatinine, uric acid, and pH detection. The detection range was 10 nM to 100 μM for creatinine, 100 μM to 500 μM for uric acid, and 4 to 9 for pH. Furthermore, interference experiments were also conducted to verify the specificity of the sensors. Subsequently, multi-channel double-sided sensing electrodes and function-integrated hardware were designed, with the standard equations of target analytes stored in the system’s read-only memory. Moreover, a WeChat mini-program platform was developed for smartphone interaction, enabling off-body detection and real-time display of target analytes through smartphones. Finally, the aforementioned electrochemical detection electrodes were integrated with the smart sensing system and wirelessly interfaced with smartphones, allowing for intelligent real-time detection in primary healthcare and individual household settings.

## 1. Introduction

Urine contains a significant amount of valuable biomarkers, including proteins, glucose, and a range of metabolites [1,2,3]. The analysis and measurement of biomarkers in urine represents a straightforward, non-invasive approach to the monitoring of an individual’s health status, the early detection of disease, the guidance of treatment, and the evaluation of nutritional status.

Creatinine is the final product of creatine and phosphocreatine metabolism in the human body. It is a valuable marker for clinical laboratory assessment of renal function, diagnosis of acute myocardial infarction, and quantitative evaluation of blood dialysis. In the human body, creatinine has two sources: endogenous creatinine is derived from muscle tissue, while exogenous creatinine is primarily derived from the metabolism of meat in the diet [4]. It has been demonstrated that the concentration of creatinine in body fluids is closely related to the kidneys, thyroid, cardiovascular disease, and muscle dysfunction [5,6,7,8]. Creatinine exists in both muscle tissue and blood and is transported to the kidneys through the bloodstream. The kidneys are responsible for filtering out a significant proportion of creatinine through glomerular filtration and excreting it in urine. The typical physiological concentration of creatinine in human urine ranges from 4.4 mM to 18.0 mM [9]. The ratio of creatinine between urine collected within 24 h and serum is an important indicator for estimating the glomerular filtration rate. The daily excretion of urinary creatinine is relatively consistent, reflecting the quality of muscle tissue in the body.

In clinical practice, traditional techniques for creatinine detection include the Jaffe colorimetric method and enzyme-based electrochemical detection. The Jaffe colorimetric method entails the reaction of creatinine in an alkaline solution with picric acid to form a red-orange chromophore, followed by the determination of the creatinine concentration using spectrophotometry. However, the colour of the creatinine-picric acid complex is similar to that of urine, which may result in potential optical interference and low selectivity of this method. Enzyme-based electrochemical detection typically requires three enzymes [10], namely creatininase, creatinase, and sarcosine oxidase, offering high specificity and sensitivity. However, this method is costly, operationally complex, and highly susceptible to limitations in enzyme stability and storage. In order to address these shortcomings, in recent years, research on non-enzymatic electrochemical creatinine sensors based on modified electrode oxidation has been increasingly conducted. In 2022, Ashakirin et al. devised a molecularly imprinted polymer comprising copper nanoparticles and poly(methyl methacrylate-co-divinylbenzene) to modify a screen-printed carbon electrode and developed a label-free and highly selective creatinine detection platform through electrodeposition [11].

Creatinine has a low electrochemical activity, so the incorporation of transition metals and metal oxides into the sensing system can improve its electrochemical activity, stability, cost-effectiveness, and ability to facilitate electron transfer reactions at low potentials. Creatinine exists in several isomeric forms, each of which possesses multiple donor groups. This allows it to engage in chelation with a range of transition metal ions, including Ag(I), Zn(II), Cd(II), Hg(II), Co(II), Cu(II), and Fe(III), thereby forming soluble creatinine-metal chelates [12,13]. This property allows for the indirect, quantitative determination of creatinine. In 2023, Teekayupak et al. employed 3D printing technology to fabricate an electrochemical sensing electrode and modified the 3D-printed electrode with copper oxide nanoparticles-ionic liquid/reduced graphene oxide composite material. The modified electrode displayed remarkable electrocatalytic activity for creatinine in the absence of enzymes [9].

Uric acid is an important biomolecule present in biological fluids such as human serum and urine and is one of the most crucial analytical indicators in clinical research [14]. It is a heterocyclic organic compound that serves as the end product of exogenous purine metabolism in animal proteins and is primarily synthesized in the liver, intestines, and other tissues such as muscles, the kidneys, and vascular endothelium. Furthermore, both living and dying cells can degrade nucleic acids, adenine, and guanine into uric acid. Typically, uric acid is metabolized through the kidneys. Uricase facilitates the conversion of uric acid into a highly soluble 5-hydroxyisourate, which subsequently undergoes degradation into allantoin and ammonia, facilitating renal excretion. However, due to genetic defects, the lack of uricase in the human body prevents the oxidation of uric acid into the more soluble compound, allantoin. The majority of serum uric acid is filtered in the glomeruli, with approximately 90% being reabsorbed, indicating significant physiological implications of uric acid. The normal range of uric acid in human serum is 0.13 to 0.46 mM, while the normal range in urine is 1.49 to 4.46 mM [15]. Changes in uric acid concentration are associated with alterations in purine metabolism, leading to various physiological disorders. Detection and quantitative analysis of uric acid in biological fluids are also related to the early diagnosis of diseases. The accumulation of uric acid in the serum can result in the formation of uric acid crystals in joints, soft tissues, and other organs, which may lead to the development of disease. Abnormal uric acid levels in the human body frequently indicate the presence of a variety of conditions, including gout, hyperuricemia, renal diseases, cardiovascular diseases, and type 2 diabetes [16,17,18,19,20].

The methods for detecting uric acid include fluorescence spectroscopy [21], capillary electrophoresis [22], high-performance liquid chromatography [23], and dual-enzyme colorimetric assay [24], among others. However, these methods have inherent limitations, including prolonged analysis times, the necessity for large sample volumes, expensive instrumentation, and issues related to sensitivity and selectivity. Therefore, there is a need to develop an economically viable, simple, rapid, and accurate method for routine analysis of uric acid concentration. Electrochemical methods in biosensing are considered the optimal approach for uric acid detection, given that uric acid itself exhibits high electrochemical active properties and is suitable for electrochemical detection. In 2021, Pan et al. developed an innovative ternary nanocomposite composed of poly(3,4-ethylenedioxythiophene) peroxide, gold nanoparticles, and electrochemically reduced graphene oxide on a glassy carbon electrode [25]. This method was employed for the simultaneous detection of dopamine and uric acid in human urine, demonstrating excellent ion selectivity and electrocatalytic activity. In 2022, Singh et al. reported a wearable electrochemical sensor for the direct measurement of uric acid in human sweat [26]. They prepared a stable electrocatalytic response using a composite of nickel and electrochemically reduced graphene oxide and detected the uric acid concentration using differential pulse voltammetry. In recent years, there has been a growing use of organic functional groups and biomolecules as immobilized matrices in electrochemical sensing electrodes. These matrices exhibit multiple active sites and excellent selectivity, sensitivity, and chemical stability, which has attracted significant attention from the scientific community. L-cysteine (L-Cys) is a semi-essential amino acid that is commonly used in the construction of biosensors. Furthermore, it offers tuneable conductivity and rapid electron switching, thereby creating a multitude of active centres for the binding of analytes [27]. The electrochemical polymerization of L-Cys has been employed as a conductive polymer in a range of electrochemical sensors [28,29].

The advent of the Internet of Things (IoT) has opened up a plethora of possibilities in the domain of healthcare, offering individuals enhanced efficiency, convenience, and personalization in their medical services. Telemedicine and remote monitoring systems represent significant applications of the IoT in the field of healthcare services, addressing the logistical challenges associated with accessing medical care. Telemedicine systems facilitate communication between medical practitioners and patients via the Internet, enabling remote diagnosis and treatment without the necessity for physical presence. Intelligent real-time monitoring devices are capable of collecting personal health data, thereby facilitating the implementation of personalized health management and monitoring strategies. The IoT has brought about significant innovations and changes in the healthcare sector, offering more efficient, convenient, and personalized medical services, while also enhancing patients’ capacity for self-management.

In this study, we utilized electrochemical analysis techniques, combined with microelectronic design and nanomaterials, to construct an integrated multi-channel electrochemical sensing system for the detection of creatinine, uric acid, and pH in urine samples. Additionally, the architecture of an intelligent medical detection system was completed, and an interactive interface for smartphones was developed. Selective sensing electrode interfaces for creatinine, uric acid, and pH were designed, and the electrochemical responses of the target analytes at different concentrations were examined using electrochemical methods. This entailed the construction of a dual-sided electrode with multiple sensing channels and the integration of associated hardware functions. Moreover, a WeChat mini-program platform for smartphone interaction was devised for off-body detection and real-time display of target analytes. Ultimately, the integration of the aforementioned electrochemical sensing electrodes with the intelligent sensing system, coupled with wireless interaction with smartphones, enables the real-time detection of analytes in scenarios such as primary healthcare and individual home use.

## 2. Materials and Methods

### 2.1. Chemicals and Materials

Creatinine (C_4_H_7_N_3_O), copper nitrate (Cu(NO_3_)_2_), Nafion perfluorosulfonic acid ion exchange resin dispersion, L-cysteine (C_3_H_7_O_2_NS), 3,4-ethylenedioxythiophene (EDOT), sodium polystyrene sulfonate (NaPSS), aniline (C_6_H_5_NH_2_), H^+^ carrier I ([CH_3_(CH_2_)_11_]_3_N), sodium tetra [3,5-bis(trifluoromethyl)phenyl]borate, dioctyl sebacate, tetrahydrofuran, gold chloride hydrate (HAuCl_4_·3H_2_O), potassium ferricyanide (K_3_Fe(CN)_6_), potassium hexacyanoferrate (II) (K_4_Fe(CN)_6_), polyvinyl butyral (PVB), polyvinyl chloride (PVC), polyvinyl alcohol butyral (C_8n_H_14n+2_O_2n_), uric acid (C_5_H_4_N_4_O), D(+)-glucose (C_6_H_12_O_6_), and vanilmandelic acid (C_9_H_10_O_5_) were obtained from Sigma-Aldrich (St. Louis, MO, USA). Phosphate-buffered saline (PBS, pH 7.4), sodium chloride (NaCl), potassium chloride (KCl), magnesium chloride (MgCl_2_), calcium chloride (CaCl_2_), and ammonium chloride (NH_4_Cl) were obtained from China National Pharmaceutical Group Corporation (China). Nitrogen-doped graphene (N-Gr) was obtained from XFNANO (China). Artificial urine purchased from BIOFOUNT was mainly composed of substances such as CaCl_2_, MgCl_2_, NaCl, KCl, phosphate, NH_4_Cl, urea, etc., but does not contain creatinine, with a pH value of 4.7.

### 2.2. Apparatus

The electrochemical modification, detection, and characterization of the multi-channel electrochemical sensing electrodes were conducted on a CH660E electrochemical workstation. The following electrochemical methods were employed: square wave voltammetry (SWV), cyclic voltammetry (CV), chronopotentiometry (CP), differential pulse voltammetry (DPV), and open-circuit potential (OCP). The morphology of the electrochemical multi-channel sensing electrodes was characterized using scanning electron microscopy (SEM) (Nova Nanosem 430, FEI, Hillsborough, OR, USA), while the morphology and structure of N-Gr were characterized using transmission electron microscopy (TEM) (TecnaiG2F20, FEI, Hillsborough, OR, USA). All electrochemical experiments were conducted at room temperature.

### 2.3. Fabrication of Multi-Channel Sensing Electrodes

**Figure 1:**
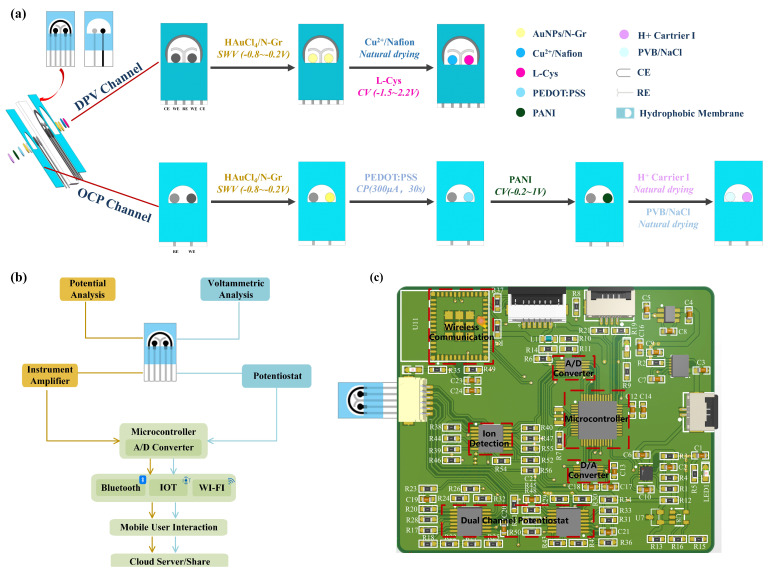
Multi-channel urine sensing system. (**a**) Schematic diagram of the modification process of dual-sided sensing electrodes for a multi-channel urine sensing system. (**b**) Schematic diagram of the multi-channel urine sensing system structure. (**c**) Multi-channel urine sensing printed circuit board and its various parts’ functions.

A double-sided multi-channel screen-printed electrode (SPE) was designed for the purpose of detecting creatinine, uric acid, and pH in urine. The electrochemical sensor was fabricated on a polyimide (PI) substrate using a multi-layer screen printing technique. One side of the sensing electrode comprised a three-electrode sensing system for simultaneous detection of creatinine and uric acid concentrations, while the other side featured a dual-electrode sensing system for pH detection. As illustrated in Figure 1a, a customized template was initially employed to print a semicircular Ag/AgCl layer on the PI substrate, forming a common reference electrode and conductive traces. Next, carbon ink was screen-printed on the PI substrate to create two circular carbon working electrodes for the individual detection of uric acid and creatinine concentrations. Subsequently, a “Y”-shaped carbon layer was printed to serve as a counter electrode, thereby forming a three-electrode system. On the other side of the electrode, a circular carbon working electrode was printed for pH detection, with a circular Ag/AgCl layer serving as the reference electrode to create a dual-electrode system. Finally, a waterproof coating was applied to the exterior of the electrodes, specifically outside the effective areas, in order to expose the detection regions. This approach concentrates the detection area, facilitating the addition and measurement of test solutions while reducing interference from other parts of the electrode. The dimensions of the electrode substrate were 3.3 cm × 1.7 cm, the diameter of the circular electrode was 1.5 mm, and the distance between each electrode was 2 mm.

### 2.4. Design of the Sensing Electrode Substrate

The design of an appropriate functionalized platform is a crucial step in the electrode modification process. Therefore, the selection of the modification material is necessary for preparing sensors with suitable applications. Gold nanoparticles (AuNPs) are extensively studied in the fields of nanoscience and nanotechnology and have found wide applications in biomedicine, materials science, and electrochemical sensors. In the field of electrochemical sensing, AuNPs have attracted considerable attention due to their biocompatibility, excellent conductivity and thermal conductivity, high chemical stability, and high surface-area-to-volume ratio [30,31,32]. AuNPs are widely used as electrode modification interfaces in the construction of electrochemical sensors, where the rapid deposition of AuNPs on various electrode surfaces can lower the redox potential of electrochemical reactions, thereby facilitating the electrochemical reaction of target molecules.

The distinctive two-dimensional planar structure of graphene endows it with a superior surface area, which is conducive to anchoring catalysts. Moreover, graphene displays high charge carrier mobility, excellent biocompatibility, and a wide potential window, thereby markedly accelerating the rates of active reactions and electron transfer between electrodes. However, due to van der Waals forces and strong π-π stacking between graphene layers, pristine graphene tends to stack together, which results in a reduction in accessible surface area and conductivity, and consequently, a decline in electrochemical performance [33]. The electronic structure of carbon-based nanomaterials can be modified through the introduction of dopants, which can be atoms or molecules. Among various doping atoms, nitrogen is the most closely related in radius to carbon and is therefore the most extensively used for doping graphene. In comparison to undoped graphene, N-Gr displays enhanced electrochemical properties, rendering it an optimal carrier for the improvement of nanocomposite efficiency. The introduction of nitrogen atoms into the graphene structure in different bonding configurations results in alterations to the electronic properties of the material. Due to its malleable electronic properties, N-Gr can be utilized as a catalyst in electrochemical systems. Furthermore, doping with nitrogen results in an increased interlayer spacing of graphene, thus enhancing the surface area and strengthening the conjugated structure [34].

Therefore, AuNPs/N-Gr can adsorb biomolecules through electrostatic interactions, exhibits rapid and efficient electron transfer kinetics, and increases the actual accessible surface area. This nanocomposite material plays a crucial role in electrochemical sensing platforms. For creatinine, uric acid, and pH sensors, a mixture of modified AuNPs and N-Gr was chosen as the sensor substrate. The specific method entailed the mixing of 1% HAuCl_4_ and 2 mg/mL of N-Gr in equal proportions, followed by the drop-casting of the resulting mixture onto the electrode surface. Subsequently, SWV was performed with a potential range of −0.8 V to −0.2 V and a scan rate of 100 mV/s for eight cycles.

### 2.5. Modification of the Creatinine Sensing Electrode

Cu(II) is an attractive alternative material with a large surface area, low cost, low toxicity, and high electrocatalytic activity, enhancing the sensitivity of electrochemical detection for creatinine. The properties of the ligand strongly affect the redox properties of copper. When using copper, the determination of creatinine is based on the chelation property of the analyte rather than its redox behaviour, as creatinine is detected due to its impact on the copper redox reaction. Exploiting this characteristic, Cu(NO_3_)_2_-modified AuNPs/N-Gr can be employed as a sensor for creatinine detection.

To ensure good mechanical stability and reliable repetitive responsiveness of Cu(NO_3_)_2_ modified on the surface of AuNPs/N-Gr electrode, Nafion was mixed into the Cu(NO_3_)_2_ solution before modification. Nafion has been proven to be an excellent candidate polymer in electrochemical modification systems, as it enhances the stability of the electrode through its exceptional film-forming ability and provides excellent mechanical and chemical stability due to its hydrophobic framework, minimizing the detachment of the modified material from the electrode surface. The specific procedure involved mixing a 10% Nafion solution with a 500 mM Cu(NO_3_)_2_ solution in equal-volume proportions, then dropping 70 μL of the Nafion-Cu^2+^ mixture onto the AuNPs/N-Gr electrode surface and letting it air dry for 4 h at room temperature (Figure 1a). Different concentrations of creatinine standard solution were dropped onto the working electrode Cu(NO_3_)_2_/AuNPs/N-Gr/SPE, and the measurements were performed using the DPV method with a scan rate of 100 mV/s over a potential range of −0.2 V to 0.3 V. The peak current responses corresponding to different concentrations of creatinine were used for calibration analysis.

### 2.6. Modification of the Uric Acid Sensing Electrode

Uric acid possesses excellent electrochemical activity, making electrochemical analysis the preferred method for its detection. The specific design scheme for the uric acid sensor involved adding 1.0 mM L-cysteine electrochemically synthesized and polymerized L-cys on the AuNPs/N-Gr sensor substrate. The synthesis was carried out using the CV method within a potential range of −1.5 V to 2.2 V, with a scan rate of 100 mV/s for 10 cycles (Figure 1a). Different concentrations of uric acid standard solutions were dropped onto the working electrode (L-Cys/AuNPs/N-Gr/SPE), and measurements were conducted using DPV within a potential range of 0 V to 0.5 V and a scan rate of 100 mV/s. The peak current response corresponding to the oxidation/reduction of uric acid at different concentrations was used for calibration analysis.

### 2.7. Modification of the pH Sensing Electrode

The pH value of urine is a critical health indicator, as it is related to the hydrogen ion concentration in bodily fluids, which reflects the metabolism and homeostasis levels in the human body. For pH sensors, proton-conductive materials play a vital role in facilitating proton transport. Membranes based on proton-conductive polymers, typically containing side-chain chemical groups, can be utilized to enhance proton movement. In chemical reactions that rely on proton transport, the overall performance depends on the transport characteristics of the polymeric proton-conductive membrane. Polyaniline (PANI) is a candidate material for proton-conductive membranes due to its low cost, high stability, ease of manufacturing, and biocompatibility. It is a commonly used medium for measuring the pH value of biological fluids.

Conjugated polymer poly(3,4-ethylenedioxythiophene)-poly(styrenesulfonate) (PEDOT:PSS) is typically synthesized through chemical oxidative polymerization, where the EDOT monomer forms cationic radicals and undergoes chain growth in the presence of an oxidant. PSS serves as a hydrophilic anionic dopant and provides a framework for the polymerization of EDOT. Simultaneously, interaction with the oxidized PEDOT results in a core-shell structure with PEDOT as the core and PSS as the shell. The sulfonate ions provided by PSS stabilize the PEDOT cations. The synthesized PEDOT:PSS exhibits excellent electrical properties, good thermal/electrochemical stability, and film-forming characteristics.

The specific design of the pH sensor was as follows (Figure 1a): A suitable amount of a mixed solution of 0.01 M EDOT and 0.1 M NaPSS was dropped onto the effective area of the pH sensor, and PEDOT:PSS was electrochemically polymerized at a constant current of 300 μA for 30 s using the CP method. Next, a suitable amount of a mixed solution of 0.1 M aniline and hydrochloric acid was dropped onto the effective area of the pH sensor, and CV was conducted for eight consecutive scans to synthesize PANI, with a scanning voltage range of −0.2 to 1 V and a scan rate of 100 mV/s. Subsequently, a H⁺ selective membrane was coated on the working electrode of the pH sensor. The preparation method for the H⁺ selective membrane involved mixing hydrogen ion carrier I, sodium tetra [3,5-bis(trifluoromethyl)phenyl]borate, PVC, and dioctyl sebacate in the proportions of 1.0 wt%, 0.5 wt%, 66 wt%, and 32.5 wt%, respectively, using tetrahydrofuran as the solvent. To maintain the stability of the potential detection, a mixture of 79.1 mg of PVB and 50 mg of NaCl was dissolved in 1 mL of methanol and applied in suitable amounts to the reference electrode of the pH sensor. The working electrode configuration was H^+^ carrier I/PANI/PEDOT:PSS/AuNPs/N-Gr/SPE. It was allowed to stabilize in standard solutions of different pH for a certain period, followed by OCP detection from −1 V to +1 V for 30 s. The average value of open-circuit potential during 30 s for different pH values was used for pH calibration.

### 2.8. Multi-Channel Sensing Electrode Integrated System

The structural design of the multi-channel urine sensing system is illustrated in Figure 1b. The designed double-sided sensing electrode served as the sample carrier, and multiple electrode interfaces were incorporated on the printed circuit board to facilitate electrochemical detection of the samples (potentiometric and voltametric analyses). Various wireless connectivity options, including Bluetooth, the IoT, and Wi-Fi, were integrated into the system. The system utilized a custom-developed WeChat mini-program to establish analysis protocols, configure signal interfaces, process data, and share results. Composition and principles of the detection device (Figure 1c): The voltage excitation for DPV was generated by the digital-to-analogue converter (DAC) module controlled by the microcontroller module. This excitation was applied to the sensing electrode through the constant potential meter module. Meanwhile, the analogue-to-digital converter (ADC) module collected the current signals generated during the chemical reaction. The measurement of OCP between the two electrodes in pH sensing was achieved through a self-built ion detection module. The measured data were transmitted and sent to the mobile application for data processing and result display via the wireless communication module. The power supply module consisted of three parts: 3.7 V to 3.3 V, 3.7 V to 5 V, and 5 V to −5 V. The 3.3 V power supply was for the microcontroller module and the wireless communication module. The 5 V power supply was for the DAC module, ADC module, and operational amplifier positive power supply. The −5 V power supply was for the negative power supply of the operational amplifier. Specifically, the microcontroller adopted the low-power STM32F103C8T6 chip, the DAC module adopted the 16-bit dual-channel low-power DAC8562 chip, and the ADC module adopted the 16-bit high-precision ADS1115IDGST chip. The constant potential meter module and ion detection module were built using the dual-power-supply operational amplifier chip AD8674, and the wireless communication module used the ESP32-C3-MINI-1.

The constant potential meter module and ion detection module are the core modules of the circuit. The constant potential meter module consisted of an inverting amplifier circuit, a non-inverting amplifier circuit, a voltage follower circuit, a transimpedance amplifier, and a reference source. It was internally connected to the DAC module and ADC module and externally connected to the sensing electrode through the reference electrode/working electrode/counter electrode interface. The voltage control loop was formed by the working electrode and reference electrode, which precisely controlled the voltage using the voltage feedback of the amplifier. The voltage at the RE terminal was controlled by the DAC module, while the bias voltage at the working electrode terminal was controlled by the reference source chip to ensure its stability. This allowed the voltage difference between the reference electrode and the working electrode to be adjusted within a certain range. The current loop was formed by the working electrode and counter electrode, and the transimpedance amplifier was used to convert the current into voltage, which was then output to the ADC module for signal acquisition. The transimpedance amplifier can also adjust the resistance to change the output voltage and thus adjust the detection sensitivity. The ion detection module utilized the characteristics of the operational amplifier, where the front-stage amplifier was connected as a voltage follower to follow the input voltage signal. Subsequently, the detection and reference signals were used as inputs to the differential amplifier circuit of the back-stage amplifier. At the same time, a summing circuit was formed by using a reference voltage to amplify the overall signal voltage. Finally, two detection signals were obtained, with the zero-voltage point being the reference voltage. The obtained detection signals need to be connected to a 1 nF filtering capacitor before being transmitted to the signal acquisition module for collecting and processing, using the ADC chip ADS1115 for measurement. The OCP detection unit detected weak voltages using a differential amplifier, and it can detect biphasic OCP from ion-selective membranes.

## 3. Results and Discussion

### 3.1. Characterization of the Multi-Channel Sensing Electrode

**Figure 2:**
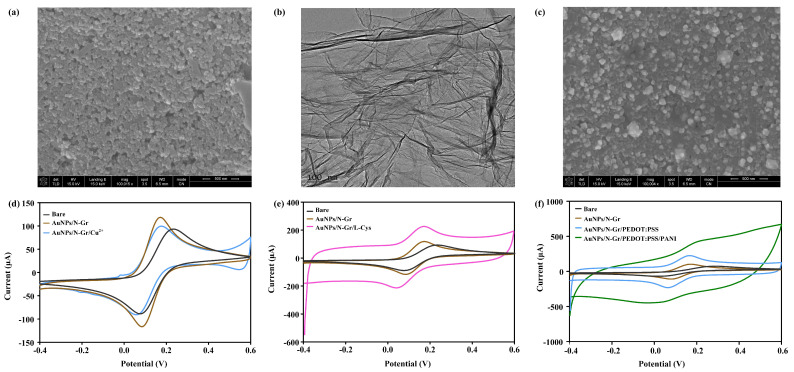
Characterization of multi-channel sensing electrodes. (**a**) SEM characterization of the bare electrode; (**b**) TEM characterization of N-Gr; (**c**) SEM characterization of the AuNPs/N-Gr electrode; electrochemical characterization of (**d**) the creatinine sensing electrode, (**e**) the uric acid sensing electrode, and (**f**) the pH sensing electrode.

Microstructural characterization of the electrode was conducted using the Nova NanoSEM 430 scanning electron microscope at a magnification of 100,000. As shown in Figure 2a, the morphology of the bare electrode appeared relatively flat. The microstructure of N-Gr was further investigated using the Tecnai G2F20 transmission electron microscope, as depicted in Figure 2b, revealing a thin, wrinkled structure, which greatly increased the surface area of the electrode. Following the electrodeposition of the N-Gr and AuNP mixture, SEM observation (Figure 2c) showed that AuNPs were embedded in the N-Gr, significantly expanding the surface area of the electrode. CV scans were performed on the sensing electrode with 100 μL of Fe^2+^/Fe^3+^ redox couple (5 mM K_3_Fe(CN)_6_/K_4_Fe(CN)_6_, 0.1 M KCL), within a voltage range of −0.4 V to 0.6 V and at a scan rate of 50 mV/s. As shown in Figure 2d, the peak coordinates for the oxidation and reduction of the electrode modified with Cu(NO_3_)_2_/Nafion were (0.177 V, 99.68 μA). The peak current slightly decreased compared to AuNPs/N-Gr but remained higher than that of the bare electrode, indicating a decrease in electrode electron transfer rate due to the poor conductivity of the Nafion membrane. This resulted in a reduced electron transfer efficiency after the modification with Cu(NO_3_)_2_/Nafion. However, the electrochemical performance of the sensor was still improved due to the modification of the AuNPs/N-Gr nanomaterial. Figure 2e presents the electrode characterization results for the uric acid sensor, where the oxidation and reduction peak coordinates for the electrode modified with poly L-Cys were (0.169 V, 226.2 μA). Compared to AuNPs/N-Gr, an increase in the oxidation and reduction peak currents indicated that poly L-Cys provided a large number of active sites for electrode reactions, enhancing the electron transfer rate. The characterization results of the pH-sensor-modified electrode are shown in Figure 2f. The oxidation and reduction currents gradually increased after the modification with PEDOT:PSS and PANI, indicating that the modified substances improved the electron transfer rate and electrocatalytic activity of the electrode, providing favourable conditions for pH sensing detection.

### 3.2. Analytical Performance of the Multi-Channel Sensing Electrode

**Figure 3:**
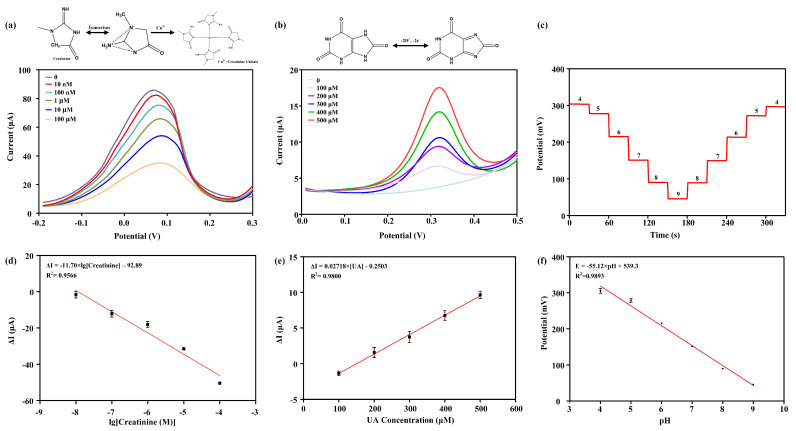
Detection results of multi-channel sensors. (**a**) Sensing detection results for creatinine, with a detection range of 10 nM to 100 μM. (**b**) Sensing detection results for uric acid, with a detection range of 100 μM to 500 μM. (**c**) Sensing detection results for pH, with forward and reverse detection in solutions ranging from pH 4 to 9. The standard fitting curves for sensing detection are shown in (**d**) for creatinine, (**e**) for uric acid, and (**f**) for pH.

The detection results of the creatinine sensor are shown in Figure 3a, with a detection range of 10 nM to 100 μM. The oxidation-reduction peak potential of DPV appeared at approximately 0.07 V, and the peak current decreased with increasing creatinine concentration. As the concentration of creatinine increased, Cu^2^⁺ bound with creatinine on the electrode surface, forming a Cu^2^⁺-creatinine chelate complex. The formation of this chelate complex reduced the concentration of free Cu^2^⁺ at the electrode surface, which in turn diminished the characteristic Cu/Cu^2^⁺ current and lead to an inverse correlation between the DPV analytical response and the increasing concentration of creatinine in the reaction system. Under these conditions, a linear regression relationship was observed between the logarithmic value of creatinine concentration and the oxidation peak current (Figure 3d). The equation of the standard fitting curve was Δ*I* (μA) = −11.70 × *lg*[Creatinine] − 7.075, with a goodness of fit (R^2^) of 0.9566, indicating excellent detection sensitivity of the creatinine sensor.

The detection range of the uric acid sensor was 100 μM to 500 μM. The oxidation-reduction peak current of DPV was located at approximately 0.32 V (Figure 3b). The peak current increased with increasing uric acid concentration. This was because uric acid is an electroactive substance, and with higher concentrations, more electrons were transferred in the electrode reaction, resulting in a larger current. Under these conditions, a linear regression relationship was observed between the uric acid concentration and the oxidation peak current (Figure 3e). The equation of the standard fitting curve was Δ*I* (μA) = 0.02718 × [UA] − 0.2503, with a goodness of fit (R^2^) of 0.9800, indicating excellent detection sensitivity of the uric acid sensor.

Figure 3c demonstrates repeated tests of the pH-ISE sensor based on PANI and H^+^ carrier I using the OCP method in PBS from pH 4 to pH 9. The results indicate that the sensor can detect pH values from 4 to 9 and complete a full cycle back to 4 with an incremental unit. As the pH increased, the H^+^ concentration decreased in the solution, leading to a reduction in the proton exchange between PANI and H^+^ carriers. Consequently, a decrease in the detected OCP was observed. Figure 3f illustrates a linear relationship between the OCP and the pH value, with the equation of the standard fitting curve being *E* (mV) = −55.12 × pH + 539.3, with a goodness of fit (R^2^) of 0.9893. The normal pH range in human urine fluctuates between 4 and 8, and the designed sensor’s measurement range can fully cover the pH detection range of human urine. H^+^ can be detected through the process of protonation with PANI and binding with H^+^ carrier I, resulting in a change in the OCP relative to the reference electrode. According to the Nernst equation, pH is defined as the negative logarithm of H^+^ activity, with a theoretical sensitivity of 59.1 mV/pH at room temperature. The actual sensitivity of the pH sensor is 55.12 mV/pH, which has a relatively small relative error and is very close to the pH sensitivity predicted by the Nernst equation.

### 3.3. Interference Study

One major advantage of electrochemical sensors is their ability to selectively distinguish and measure target substances with specificity. Selectivity is a crucial factor when these sensors are used for measuring creatinine, uric acid, and pH in complex media such as human urine. Therefore, it is necessary to investigate the influence of these major analytes on the sensor’s performance. To evaluate the specificity of the sensor, several substances that could potentially interfere with the analysis in bodily fluids were selected for anti-interference experiments. For the creatinine sensor, anti-interference experiments were conducted with 1 μM glucose, lactate, uric acid, vanilmandelic acid, and NaCl using the same measurement method as creatinine detection, which was DPV, with an electrode potential range of −0.2 to 0.3 V. For the uric acid sensor, anti-interference experiments were conducted with 300 μM NaCl, glucose, creatinine, vanilmandelic acid, and lactate using the same measurement method as uric acid detection, detecting the DPV response of interfering substances within the potential range of 0 to 0.5 V. For the pH sensor, PBS with a pH of 7 was dropped onto the electrode, followed by the addition of interfering ions with physiological relevant concentrations of 10 mM NaCl, 0.5 mM CaCl_2_, 1 mM NH_4_Cl, 0.5 mM MgCl_2_, and 1 mM KCl to the PBS solution with a pH of 7. After waiting for a stable period of 10 s, the interfering substances were measured using the OCP method.

**Figure 4:**
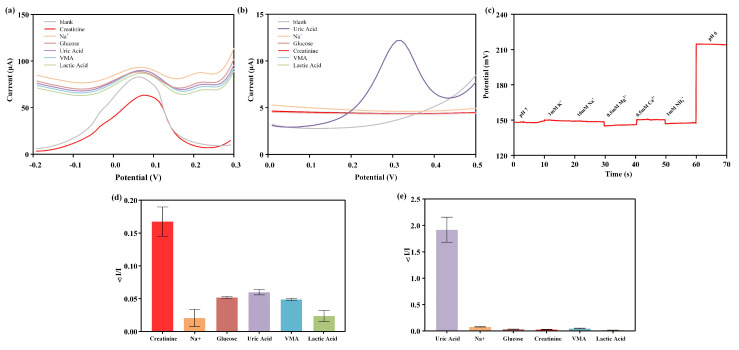
Interference test of multi-channel sensors. (**a**) DPV curve of the creatinine sensor for detecting different interferents. (**b**) DPV curve of the uric acid sensor for detecting different interferents. (**c**) OCP curve of the pH sensor detecting different interferents. Bar graph of the interference test signal for the creatinine sensor (**d**) and uric acid sensor (**e**).

Figure 4a–c represents the DPV curves of different interferents in the creatinine sensor interference test, the DPV curves of different interferents in the uric acid sensor interference test, and the OCP curves of different interferents in the pH sensor interference test, respectively. Figure 4d,e show the relative changes in electrochemical response of different interferents in the interference testing of creatinine and uric acid sensors. The results indicate that the relative change in electrochemical response caused by interfering substances was significantly smaller compared to the changes induced by the target molecules (creatinine, uric acid, and H^+^). This indicated that the designed creatinine, uric acid, and pH sensors exhibited specific responses to the measured substances, making them suitable for the analysis of creatinine, uric acid, and pH in urine.

### 3.4. Artificial Urine Analysis and Mini-Program Display

**Figure 5:**
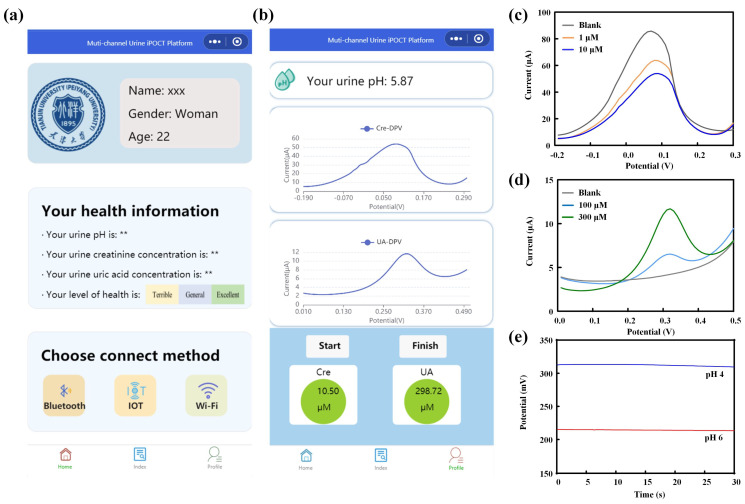
(**a**) WeChat mini-program interface. (**b**) Displayed results of multi-channel sensing detection on the WeChat mini-program. Sensing detection results of creatinine (**c**), uric acid (**d**), and pH (**e**) in artificial urine.

To validate the applicability of the multi-channel sensor in detecting the pH value and the concentrations of creatinine and uric acid in real samples, we employed a spiked artificial urine method for testing. During the detection process, the detection curves were displayed in real time via a WeChat mini-program, and the concentrations of creatinine and uric acid, as well as the pH value of the samples, were calculated based on standard equations (Figure 5a,b). Specifically, solutions containing 1 µM and 10 µM of creatinine, along with 100 µM and 300 µM of uric acid, were prepared using the spiked artificial urine method, and the pH of the samples was adjusted to 4 and 6, respectively. The pH value of the samples was initially determined using the pH sensor, followed by a 10-fold dilution of the artificial urine samples with 1 M PBS solution for the detection of creatinine and uric acid concentrations. In order to ascertain the concentrations of creatinine and uric acid, the current response of the sensing electrode to a solution of blank artificial urine was initially measured. This was followed by the measurement of the current response to a solution of spiked artificial urine. The discrepancy between the two measurements was designated as ΔI and subsequently incorporated into the sensor’s standard equation. As illustrated in Figure 5c–e, the multi-channel sensor demonstrated effective detection of artificial urine samples with pH values of 4.12 and 5.87, containing 0.89 µM and 10.50 µM of creatinine, as well as 94.6 µM and 298.72 µM of uric acid, respectively.

## 4. Conclusions

In this study, we utilized electrochemical analysis techniques in conjunction with microelectronic design and nanomaterials to construct an integrated multi-channel electrochemical sensing system and developed an intelligent medical detection system. The electrochemical sensing detection of creatinine, uric acid, and pH was investigated. By electrochemically modifying the SPE, we measured the DPV responses of creatinine and uric acid at different concentrations, as well as the OCP response of pH, establishing the standard electrochemical sensing detection equations. We designed double-sided multi-channel sensing electrodes and integrated them with a printed circuit board, allowing interaction with a WeChat mini-program on a smartphone to achieve intelligent real-time detection of creatinine, uric acid, and pH in urine.

The currently designed creatinine and uric acid sensors have a detection range at the μM level. Considering that the normal physiological ranges for creatinine and uric acid are at the mM level, samples still require dilution for detection. Therefore, we plan to further optimize the detection range and simplify the detection process in future research. Additionally, the functionality of the WeChat mini-program designed to complement the sensor has certain limitations. Future studies will focus on developing a mobile application platform to enable personalized testing in non-clinical environments, such as individual and home use. We envision that this smartphone-based mobile biosensing tool will enable daily and comprehensive monitoring of metabolites in the future, thereby transforming clinical diagnosis into a non-clinical setting for optimal and personalized treatment of metabolic disorders.

## Data Availability

Dataset available on request from the authors.
